# LagunAR: A City-Scale Mobile Outdoor Augmented Reality Application for Heritage Dissemination

**DOI:** 10.3390/s23218905

**Published:** 2023-11-01

**Authors:** Isabel Sánchez Berriel, Fernando Pérez Nava, Pablo Torres Albertos

**Affiliations:** Departamento de Ingeniería Informática y Sistemas, University of La Laguna, 38200 San Cristóbal de La Laguna, Spain; fdoperez@ull.edu.es (F.P.N.); pablotorresalb@gmail.com (P.T.A.)

**Keywords:** cultural heritage, location-based mobile augmented reality, 3D reconstruction

## Abstract

In this paper, we introduce LagunAR, a mobile outdoor Augmented Reality (AR) application for providing heritage information and 3D visualization on a city scale. The LagunAR application was developed to provide historical information about the city of La Laguna in the XVI century, when it was the main city in the Canary Islands. The application provides a reconstructed 3D model of the city at that time that is shown on a mobile phone over-imposed on the actual city using geolocation. The geolocated position is used also for providing information of several points of interest in the city. The paper describes the design and implementation of the application and details the optimization techniques that have been used to manage the full information of the city using a mobile phone as a sensor and visualization tool. We explain the application usability study carried out using a heuristic test; in addition it is probed by users in a qualitative user test developed as preliminary research. Results show that it is possible to develop a real-time application that shows the user a city-scale 3D model and also manages the information of the points of interest.

## 1. Introduction

Dissemination of cultural heritage (CH) is a fundamental aspect of heritage management, along with research, protection, and conservation. The goal of dissemination is to promote a favorable attitude towards heritage, fomenting its identification, appreciation, preservation, and enjoyment by the public.

There are various ways to disseminate the historical heritage of a location. In the past, people relied on written records or stories from its contemporary inhabitants. Later, the use of drawings, photographs, or even videos have been used to obtain information about historic locations. However, personal visits to historic places with the company of expert guides often leave a more profound impact. Even the presence of historical plaques at the site can provide a valuable repercussion.

Advances in technology have opened new opportunities for virtual visits to places in the past, through digital reconstructions of historic locations and the use of digital guides or digital recreations of its inhabitants. The integration of these new technologies with mobile devices offers an engaging and accessible means of promoting CH to the general public [1,2]. They also contribute to the preservation of CH from degradation [3].

In this paper, we will present LagunAR, an Augmented Reality (AR) application for the dissemination of the historic heritage of the UNESCO World Heritage city of San Cristóbal de La Laguna (Spain) in the XVI century. The application provides a reconstructed 3D model of the city at that time that is shown on a mobile phone over the actual city using geolocation. The geolocated position is used also for providing information of several points of interest in the city and its inhabitants. 

Our research is driven by a dual-fold motivation. Firstly, to collaborate with the city council of San Cristóbal de La Laguna in their efforts to enhance heritage conservation through the utilization of new technologies. Secondly, our research is geared towards tackling the intricate technical challenges inherent in the creation of real-time AR applications at a citywide scale, with a particular emphasis on optimizing performance and enhancing the user experience on mobile devices.

Augmented Reality (AR) [4] is a technique that combines real and virtual content, provides a real-time interactive environment, and registers them in 3D. In order to develop an AR application, there are four essential elements that must be addressed: tracking, virtual environment modeling, visualization and interaction [5,6]. 

Tracking is necessary to align the virtual content with the real environment. It involves monitoring the position and orientation of the AR device and the virtual content using sensors, cameras, and computational techniques to maintain the correspondence of the virtual content with the real-world environment. There are various kinds of tracking methods, including camera-based, sensor-based, and hybrid methods. Camera-based tracking may be based on recognizable pre-determined markers or using generic geometric features from the real environment. 

Sensor-based tracking uses inertial sensors such as gyroscopes and accelerometers to determine the position and orientation of the AR device. The gyroscopes are used to measure the angular velocity to detect orientation, while the accelerometers measure the gravitational acceleration to detect the movements of the device. The integration of both sensors allows for an accurate tracking of the device’s pose. This tracking method is cost-effective and provides high update rates but suffers from positional drift due to the accumulation of small measurement errors from the accelerometer and the gyroscope. Location-based tracking, on the other hand, relies on the Global Navigation Satellite System (GNSS) sensor to access information about the device’s location [7]. Recently, the widespread use of mobile devices that integrate Inertial Measurement Units (IMU) and GNSS sensors has accelerated the developments of Mobile Augmented Reality (MAR) applications.

Finally, hybrid tracking, which is a combination of the above tracking methods, can yield better results than when each method is employed separately. In particular, inertial tracking is often combined with other tracking methods in the heritage domain [8].

The second element to be addressed by an AR application is virtual content modeling. This process simulates real objects and their state in virtual space, the behavioral rules that the objects obey, and relationships and interactions between them [9]. These objects may be obtained using actual measurement, geometric modeling, or artificial construction. Actual measurement refers to the object data acquired through the processes of 2D or 3D scanning [10], geometric modeling refers to the creation of virtual environments using algorithms and mathematical processes like procedural techniques [11], and artificial construction refers to object data that is completely fictitious [12]. Once the object information is acquired, the complete modeling depends on its properties [9]: its spatial structure and its physical, behavioral, and dynamic features. 

After the definition of the tracking and modeling elements, an AR application must address the visualization of the modeled objects. The goal is to create an immersive experience where virtual objects appear to exist in the physical space, allowing the user to interact with them in real time. The visualization process is usually divided into three distinct layers: the real world, the augmented 3D world, and screen space [5]. The real world is used as a background and is captured by a camera in real time. The augmented 3D world is the place where virtual objects appear, overlayed in the real-world environment. Finally, the screen space is where the 2D application interface displays the preceding layers and interactions occur [13]. There are several techniques employed in the visualization of virtual objects, including 3D object visualizations, annotations and labels, or assisting visual aids. 

Finally, the last element that defines an AR application is interaction. The goal is to provide intuitive and natural interfaces for the user [6,8]. In general, there are six possibilities for interface selection. Tangible interfaces [14] utilize physical objects as the means of interaction with the virtual environment. Physical objects serve as controllers, allowing users to manipulate virtual elements in the AR space. Collaborative interfaces [15] enable multiple users to interact with AR content, together promoting teamwork and cooperation. Device-based interfaces [5] are typically centered around the use of a specific device, such as a smartphone or tablet, to interact with the virtual environment. The device serves as the primary means of interaction with the AR content. Sensor-based interfaces [10] use accelerometers, gyroscopes, or GNSS to track the position and orientation of the AR device and the virtual elements in real time. Multimodal interfaces use multiple modes of interaction, such as touch, gesture, voice, and vision, for the user to interact with the virtual environment. Finally, hybrid interfaces [16] combine several forms of interaction, such as device and sensor-based interfaces, to create a more immersive AR experience. 

Because most users have never experienced AR applications, it is important to analyze the usability of the application. This question can be addressed using different approaches that we can group into three main categories: user tests, as in [17,18], or qualitative tests, such as those used in case study 2 in [18], and finally heuristic evaluation performed by experts, as in [19]. User tests are quantitative techniques that require recruiting enough number of users to guarantee the reliability of the results. Qualitative user testing is based on tasks performed by a small group of users while a facilitator monitors them by somehow recording the way that the user interacts with the application. These types of tests allow the detection of design errors; they are very useful in the initial stages of development to correct them in the early phases of the project life cycle as a preliminary task. Finally, it is possible to use evaluations carried out by experts, which check a collection of factors that determine in advance potential usability problems that the application could have.

This paper is structured into six sections. Section 2 offers an overview of related works in the field of Augmented Reality (AR). Section 3 details the methodology employed in the development of our application. Section 4 presents the outcomes of our approach, while their implications are discussed in Section 5. Finally, Section 6 provides a comprehensive summary of our research conclusions.

## 2. Related Works

The field of AR has experienced a constant evolution in recent years and several surveys of AR [5,20,21] and MAR [22,23] detail the advances in the field. The goal of our project is to disseminate the historical heritage of the city of San Cristóbal de la Laguna in the XVI century using a 3D model of the city in a Location-Based MAR application (LBMAR) [22]. Therefore, in this section, a brief overview of AR systems for CH is included. As detailed in [5], the application areas of AR in CH can be divided into education, exhibition enhancement, exploration, reconstruction, and virtual museums according to the primary goal of the application. 

The main goal of AR in education is to enhance the learning experience by providing students with an interactive, immersive, and engaging environment [24,25]. Another aspect of AR in CH is to enhance the visitor experience at physical museums and exposition sites [26]. This is achieved by using AR technologies to guide visitors through exhibitions, providing them with additional information and enhancing their overall experience [27]. Exploration is another key aspect of AR in CH, as it allows one to enhance the visitor’s experience and provide them with a more interactive and informative way of exploring historical sites [28,29]. Reconstruction applications display reconstructed views of tangible and intangible CH [5]. Such applications allow users to visualize CH assets that existed only in the past or that partially exist nowadays. Reconstructed assets can be presented in three forms: tangible, intangible, and a blend of both. Finally, AR technology can be used to create virtual museums, which simulate and present tangible and intangible assets in digital forms to the public. Some of these areas overlap. For instance, an AR reconstruction application might also allow a user to learn the history of the reconstructed site or enable it to be used in an exhibition. 

A common requirement for all those applications is the necessity to engage the user. The integration of gamification principles within Augmented Reality (AR) applications in the context of heritage offers a way to retain the user’s attention [30,31,32,33]. By incorporating game elements such as interactive challenges, quests, and rewards, AR heritage experiences can transform passive onlookers into active participants, fostering a deeper connection with historical narratives and cultural artifacts [34]. Gamification not only enhances user engagement but also encourages exploration, knowledge retention, and a sense of accomplishment, ultimately contributing to more vibrant and immersive heritage preservation and dissemination efforts. 

Since our project can be framed into 3D reconstruction applications [35], we present some examples of AR reconstruction applications centered around 3D objects [36]. From its beginnings, AR has been used to recreate cities, historical buildings or monuments. In [37], a group of 3D buildings of Verona (Italy) in Roman times were reconstructed for its use in an AR educational application. We can also find examples of the reconstruction of individual buildings. The Basilica of Sant’Ambrogio in Milan was reconstructed in [38], starting from the 3D survey and the data collection of the historical records of the church to develop an AR experience that improves interactivity. In [39], a 3D reconstruction of the Nerva Forum in Rome was recreated over several real-world elements. A 3D model roman house was reconstructed in [20], including its interior space, to recreate the life of Ovid, an ancient Roman poet. In [7], a 3D reconstruction of the Portuguese Malacca in Melaka city (Malaysia) was used with world illumination to create realistic virtual AR scenes. A 3D representation of the disappeared convent of El Carmen in Logroño (Spain) was reconstructed in [40] and positioned using AR over a printed plan using a mobile device. In [41], a 3D model of Sheffield’s medieval castle was virtually restored in the Castlegate area in Sheffield (United Kingdom) where it once stood. 

We can also find some partial reconstructions to complete existing historic elements. In [42], the Baroque vault of the Cathedral of Valencia (Spain) was reconstructed and the existing reredos was also augmented by means of a replica of its former Renaissance silver relief interior panel. A 3D reconstruction of the Northern door of the Aurelian Wall at Castra Praetoria was used in [43] to augment the remains of the wall. In [44], a 3D outside sculpture was virtually located over the Black Church in Brasov (Romania). 

There are several examples of the recreation of historic sites by inserting 3D elements. In [45], AR technologies were used in the Piazza Garraffo in Palermo (Italy) to virtually insert its baroque fountain, originally placed there. Another example is the Vivitica project, which was presented to reactivate the Cisneros Marketplace at Medellín (Colombia) where a 3D representation of the site was included in an AR application. In [46], a 3D reconstruction of Bergen-Belsen Nazi concentration camp was presented to offer a localized virtual immersion in the place in 1944. 

A city-scale AR recreation can be found in [47], in which the CityViewAR project was presented. The application was developed to provide geographical information about the city of Christchurch (New Zealand), which suffered several earthquakes in 2010 and 2011. The application had information and 3D models of more than 100 buildings. 

Archeological sites have also been reconstructed to provide an idea to the public of what they might have looked like. A mobile AR application was developed in [48], where 3D models of some Apulian archaeological areas (Italy) were combined with multimedia content to allow visitors to fully understand the history and the features of the ancient sites. In [49], digital models of underwater archeological sites were used with AR tools, allowing archaeologists to study the virtual site from within. 

AR reconstruction applications with 3D models have also been used to enhance museums and exhibitions. Several examples were presented in [50]: a 3D reconstruction of roman buildings over a map of the Forum Romanum in Rome, a 3D model of the Berlin wall overlayed to the city map or a 3D reconstruction of the first temple in Satricum (Italy) that was used to augments today’s photographs. In [51], the ARCO system, which allows museums to build and manage Virtual and Augmented Reality exhibitions based on 3D models of artifacts, was presented. Digitized virtual objects in the system are stored in a repository, from which the application makes a selection depending on a query and renders it to the 3D environment or superimposes it on a marker. The problem of situating large artifacts on the limited space of a museum is addressed in [52]. To overcome space limitations, an AR approach based on 3D models was proposed and demonstrated with several Roman artefacts found in Modena (Italy). In [53], a mobile augmented guide for Casa Batlló museum in Barcelona (Spain) with artificially constructed 3D virtual animated models was presented.

Finally, AR can also be used to create Virtual Reality tours of historical sites. The Archeoguide project was first presented in [8] using AR for personalized tours in cultural heritage sites. The system provided 3D reconstructions of ancient ruins that were integrated with images streamed from a webcam. Along the same lines is the Lifeplus project [54]. This project recreates the archaeological site of Pompeii (Italy), inserting in real scenarios of the disappeared town a recreation of Roman flora, fauna and daily life in 3D. In [55], three 3D reconstructions of historical buildings in the city of Chania (Greece) were overlayed onto the real world while users hold their mobile phones walking on-site. Another mobile phone application is the New Philadelphia AR Tour [56] that allows the visitor to walk through several historical 3D building reconstructions using AR.

The preceding works show several benefits of the use of AR in CH:

1. Enhanced engagement and learning: AR can make heritage experiences more engaging and interactive. Users can explore historical sites, artifacts, and information in a visually immersive manner, leading to increased interest and learning about the heritage.
2. Preservation and accessibility: AR can aid in the preservation of heritage sites and artifacts by reducing physical deterioration due to increased visitor traffic. Additionally, it can make heritage accessible to a wider audience, including people with physical disabilities, by providing virtual tours and information.
3. Dynamic and personalized experiences: AR allows for dynamic and personalized experiences. Users can choose the information they want to access, customize their tours, and view historical reconstructions, helping them connect with the heritage in a more meaningful way.

They also show several drawbacks of the use of AR in CH:

1. Complexity of development: developing and implementing AR applications is complex and often requires specialized expertise.
2. Accessibility: not all visitors may have access to the required technology, such as smartphones or AR headsets.
3. Usability challenges: While AR enhances the user experience, it can also introduce usability challenges. Users may face issues related to tracking accuracy, or interface design, which can detract from the overall experience and frustrate visitors.

These benefits and drawbacks should be carefully considered when integrating AR into CH contexts, and a balanced approach is essential to ensure that the technology enhances rather than detracts from the overall heritage experience.

In this paper, we will present some new contributions to the use of AR in heritage that can be summarized as follows:

1. Development of the LagunAR application: The paper introduces and details the development of the LagunAR application, which offers users a reconstructed 3D model of the whole historical center of San Cristóbal de La Laguna in the 16th century. This application enables users to navigate the whole city while providing information about points of interest, enhancing the engagement with the city’s heritage.
2. Technical Evaluation: The paper presents a comprehensive technical evaluation of the LagunAR application. This evaluation includes assessing location and angle precision, indicating that the application delivers accurate location tracking within acceptable limits. This information is critical for understanding the performance of the application and its potential for further enhancements.
3. Performance metrics for mobile AR: The paper evaluates the performance of the LagunAR application on mid-range smartphones using various metrics, including frame rate, CPU usage, GPU usage, triangle count, and memory usage. The results demonstrate that the application offers a smooth and engaging user experience, with stable frame rates and efficient CPU and GPU utilization.
4. Usability assessment: The paper assesses the usability of the LagunAR application through both heuristic evaluation and qualitative user tests, conducted with individuals unfamiliar with AR technology, with the purpose of detecting design issues. The usability expert’s evaluation highlights the positive aspects of the interface design and functionality while also providing suggestions for improvement. The preliminary studio with users yields insights into the application’s ease of use and identifies areas for enhancement, such as addressing tracking accuracy and improving text readability.

These four contributions collectively provide a comprehensive understanding of the LagunAR application’s development, technical performance, and usability, offering valuable insights for the development of similar city-scale AR outdoor applications with georeferenced 3D models.

## 3. Materials and Methods

The development of a location-based mobile AR application involves several crucial steps, which were been considered in the design process of the LagunAR application. The following is a detailed description of these steps:

1. Definition of the user requirements and target audience: In our case, as the goal of the application is to disseminate the CH of the city, the audience must be as general as possible while keeping user requirements in terms of hardware and mobile usage knowledge to a minimum. However, we expect that the profile of potential application users corresponds to Archaeologist in the harmonized typology ACUX for classifying cultural destination users by their characteristics and preferences. This kind of user visits cultural spaces to know the ancient history, the cultural heritage and the archaeology of the destination [57].
2. Analysis of the AR application. We had to define the main elements of the application: tracking techniques, virtual environment modeling, visualization methods and interface design.
3. Select the development platform and tools: In this step, we studied the development platforms that support location-based AR and integrate with GNSS and IMU for orientation. We also selected some external tools to support the development of 3D models, AR content, and user interaction.
4. Implementation of the application:
  1. Modeling of the virtual environment: We had to create or import the 3D models and AR content to use in the application. They had to be optimized for mobile devices.
  2. Implement location-based tracking: The techniques to use GNSS and IMU to track the user’s location and orientation in real-time were developed. We had to combine the tracking system with the 3D models and AR content to provide an immersive experience.
  3. Visualize AR content: What graphics optimization techniques to use to render the 3D and AR content in real-time. The use of lighting, shading, and other visual effects to enhance realism.
  4. Add user interaction: How to design a friendly user interaction interface to enable users to interact with the AR content. To study the incorporation of other communication modalities like voice to make the AR experience more engaging and interactive.
5. Testing and refinement of the application: Test the application with real users to evaluate its usability and performance.

**Figure 1:**
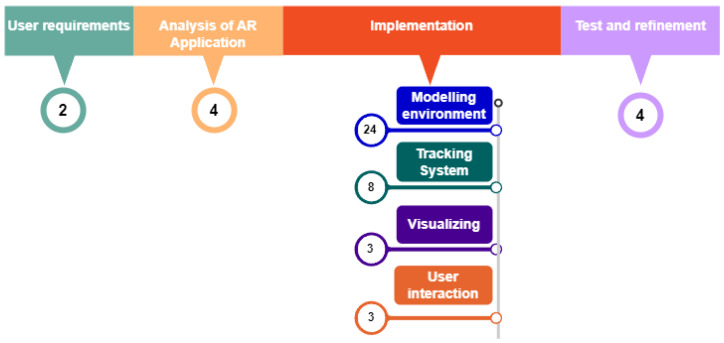
Flowchart of the methodology with time spent in the tasks.

Figure 1 shows the flowchart of the followed methodology. The number means time in weeks employed in each one of them. The time spent on the project was 48 weeks, allocated as follows: 2 weeks for specifying user requirements, 3 weeks for analysis of the AR application, 48 weeks employed in the implementation and 4 weeks for testing and refinement The implementation phase required the main effort in the project, so we have detailed the subtasks with their cost in time.

Once the requirements of the application were defined and the analysis of the AR application was completed in steps 1 and 2, it was necessary to choose a development platform, as stated in step 3. This is detailed in the following section.

### 3.1. Development Platform and Tools

In our case, we selected Unity [58] to implement the AR application. This is a common game development engine that has several desirable features: cross-platform development, the C# programming language that is widely used in the game development industry, a large community of game developers and a significant library of resources that can be used to enhance applications. 

Besides those general features, there are several specific reasons why Unity was selected to implement this application:

1. Modeling of the virtual environment:

Unity provides a variety of tools for creating 3D models, including support for importing objects from other 3D modeling software like Blender. Unity’s rendering engine allows the creation of realistic lighting and shading effects and supports physical-based rendering (PBR). It also provides tools for optimizing 3D models for performance, including the use of mesh simplification, texture compression and Level of Detail (LOD) management.

2. Tracking:

Unity provides built-in support for various location and orientation sensors, including GNSS and IMU, facilitating the implementation of tracking procedures that utilize these sensors.

3. Visualization

Unity provides support for shaders and visual effects, allowing the development of visually appealing AR experiences. It also provides tools for graphics rendering optimization like occlusion culling, which reduces the number of objects that need to be rendered by the mobile device, improving performance.

4. Interaction

Unity provides a variety of User Interface (UI) components, making it easy to create interfaces that are friendly and easy to use. It also provides tools for implementing commonly mobile-used gesture-based interaction, including support for touch, swipe, and pinch gestures. 

We now proceed to detail the main elements of the application: virtual environment modeling, tracking, visualization, and interaction. 

### 3.2. Modeling of the Virtual Environment

In this section, we will describe the reconstruction of the city that has been used in this project [59]. 

#### 3.2.1. The City of San Cristóbal de La Laguna at the End of the 16th Century

**Figure 2:**
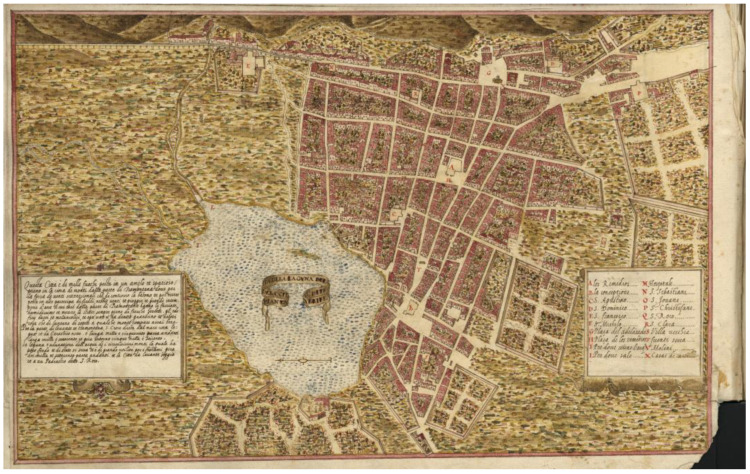
The map of San Cristóbal de La Laguna by Leonardo Torriani in 1588 [60].

The city of San Cristóbal de La Laguna is located in the island of Tenerife in the Canary Islands (Spain). It was founded in 1496 by the “Adelantado” (Royal Commissioner) Alonso Fernández de Lugo. It was located on a plain meadow surrounded by hills that provided natural defense. The surrounding forests and lagoon made the city an ideal location for settlers. The first known map of the city was made by the Italian engineer Leonardo Torriani in 1598 (see Figure 2) [60]. He described a city with a “thousand houses, each with a large yard filled with orange and other beautiful trees”. Torriani described several singular buildings (Cabildo’s house, churches, convents, hospitals) in the legend of the map. The fields outside the city were used for communal pastures and farmland.

#### 3.2.2. Historical Documentation

The main elements considered in the 3D reconstruction of the city were its houses, singular buildings (administrative constructions, churches, convents, and hospitals), other city elements like fountains or bridges, and its inhabitants [59]. The 3D modeling encountered many difficulties due to the limited remains of buildings at that time that had survived without major changes. Therefore, several forms of documentation were utilized including historical archives, photographs, sketches, and other maps. 

In [61], five different types of architecturally functional houses are described in the city at that time, based on the economic activities. These typologies can be identified based on the distinct composition of their facades, doors, windows, and roofing. The five types are “terrera” (one-floor house), “granero” (the house-barn), “sobradada” (several floors house), “commercial” (the commercial house), and “armera” (armory house). The terrera is a one-floor house that varied in features depending on the owner’s economic level and is located in the oldest part of the city. The granero is a two-floor house with the upper floor intended for cereal storage. A sobradada is usually intended for the landowner’s residence. The comercial is a two-story house with a mezzanine, facilitating public access for commercial reasons. Finally, the armera is located on the main streets as a sign of the importance of its inhabitants, and some examples have stonework on the facade that frames doors and windows (see Figure 3 from [62]).

The reconstruction of the singular buildings of the city was hypothesized from indirect sources, since the map only shows its ground outline. It is known that the singular buildings that Torriani depicted in 1588 have undergone many modifications over the centuries, making their current state different from their original form. Their reconstruction was based on historical research, supported by documentary, graphic, and historiographical sources. When the documentation was insufficient, other contemporary images were used to make approximate proposals, or the configuration was replicated from the current building.

To position the houses, singular buildings and other city elements like fountains or bridges in geographical coordinates, a Digital Elevation Model (DEM) was used. This poses several problems, since the true elevation of the terrain at the time is not available and there have been significant changes to the terrain over time, including the desiccation of the city lagoon in 1839 and the construction of various city elements over small ravines. To address these challenges, an up-to-date elevation was used which was processed to reflect the terrain as it was in 1588. 

Finally, to provide a realistic city reconstruction, several characters from different social strata were created. There are no direct sources to study the look of the city inhabitants at that time, since there are no examples of complete garments, so the main reference regarding clothing used in Spain at that time was used [50], along with several pictorial works in those years.

#### 3.2.3. The 3D Model of the City

In [55], the CityEngine (CE) software is used to generate the 3D model of the city. This software utilizes a Computer-Generated Architecture (CGA) shape grammar that can be further detailed by incorporating external 3D models. 

**Figure 4:**
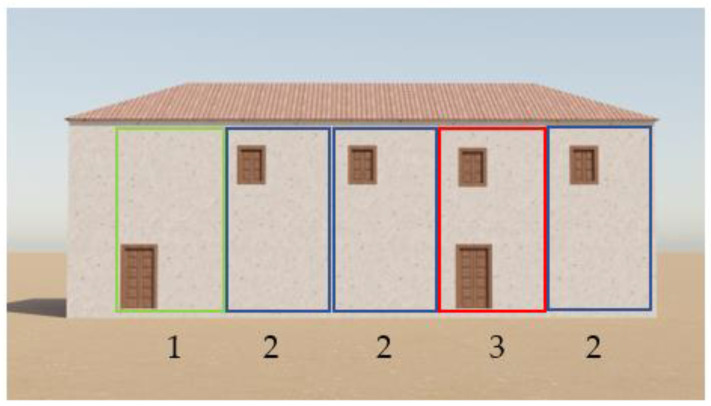
Building blocks used to model the facade door (number 1 in green), window (number 2 in blue), and door and window (number 3 in red).

The use of 3D models is resource-intensive and impacts the applications performance, particularly on mobile devices. It is important to optimize the 3D models for mobile devices by reducing their complexity, minimizing their polygon count, and optimizing textures and materials. In our case, due to the large area of interest, covering approximately 15 km^2^ and containing around 1300 procedurally generated houses and 16 singular 3D buildings, the houses in the model were kept simple with minimal geometric detail. All the houses were generated generatively resembling the houses in Torriani’s map from three simple building blocks, as shown in Figure 4.

**Figure 5:**
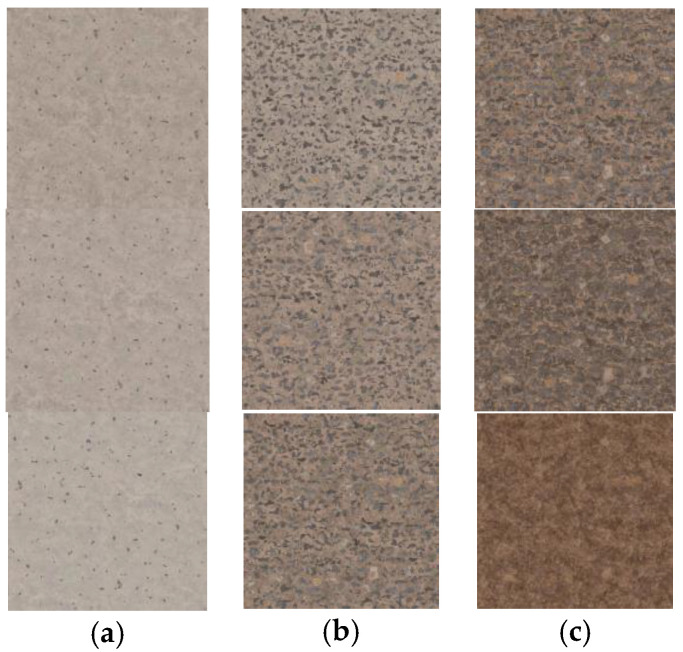
Set of façade textures used to model the houses and singular buildings [59]. (**a**) Three textures for houses in the central part of the city, (**b**) Three textures for houses in the intermediate part of the city, and (**c**) Three textures for houses in the periphery of the city.

To compensate for the lack of geometric detail, high-quality textures were applied to the models. Texturing techniques, such as the use of Physically Based Rendering (PBR) materials, have been used to describe the visual properties of a surface in a physically plausible way, to obtain realistic results under all lighting conditions. Additionally, UV mapping techniques have been employed to ensure the textures are applied consistently and correctly to the 3D models. To optimize the textures, only a limited set of them were used for each of the components in the house—façade, woods, or roofs—and each of the zones of the city—central, intermediate of periphery—as seen in Figure 5.

All the house models were exported from CE to Blender, where they were integrated with the rest of the elements in the city. 

**Figure 6:**
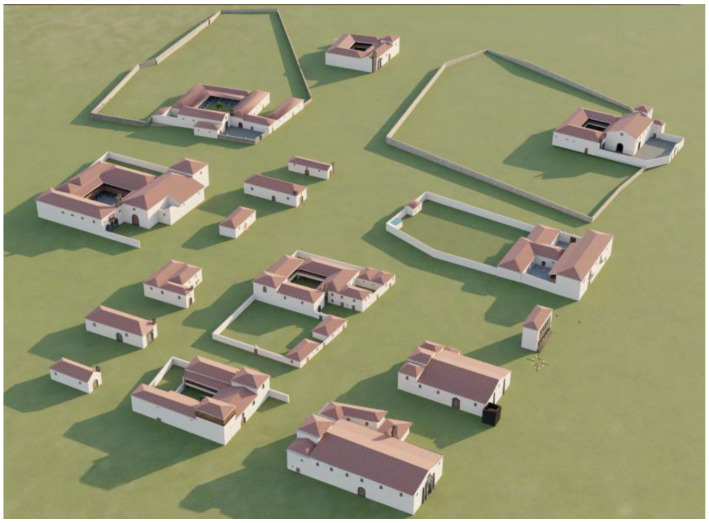
Set of singular buildings.

The 3D models of the singular buildings were generated using the 3D/BIM software Edificius. It involved the creation of 16 singular buildings of varying complexity from small churches to complex administrative buildings, as shown in Figure 6.

The generated models were exported from the Edificius software to Blender, where they were simplified and corrected. Most of the textures of the singular buildings were shared with the textures of the houses to improve the performance of the application.

**Figure 7:**
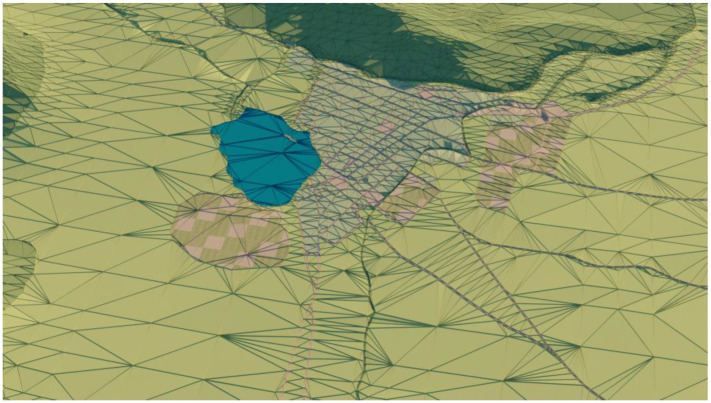
Triangulation of the original DEM.

The original DEM was found to be too dense for practical applications. To simplify the DEM, a recursive process of triangulation was used. Triangulation involves breaking up the surface of the DEM into smaller triangles, which can be used to create a simplified 3D mesh for visualization. It is a recursive process in which the DEM is continually refined by adding more triangles until it is simplified enough for use on a mobile phone. The result of this process can be seen in Figure 7, which shows a simplified 3D model of the city’s landscape that can be viewed on a mobile phone.

The next phase in the city’s reconstruction involved modeling the elements depicted on the map [59]. This process entailed digitizing a series of 2D layers, capturing the positional and the geographical distribution of various features within the city and its surroundings, including blocks, streets, gardens, plots, the lagoon, meadows, and more. These 2D map layers were subsequently transformed into 2D geographical coordinates using a georeferencing technique [36], aligning the digitized image of the map or aerial photo with a geographic coordinate system. By identifying a set of common reference points shared by both coordinate systems, signifying their geographical equivalence, 563 Ground Common points were designated on the map, including the block and crop polygons [63]. Then, the corresponding pairs from the historical map were located within modern cartography with the aid of OpenStreetMap data [64] and the PNOA orthophoto for the city [39]. These points were triangulated to establish a piecewise affine transformation linking the map to the geographic coordinate system. Once this transformation was established, it became feasible to represent the 2D layers from the historical map within the geographic coordinate system. Although the accuracy of the block and crop layers was optimal, certain manual post-processing was necessary to refine specific layers, such as introducing paths leading out of the city or adjusting elements related to mountains or ravines. For these adjustments, reference was primarily drawn from other city plans and the PNOA orthophoto.

**Figure 8:**
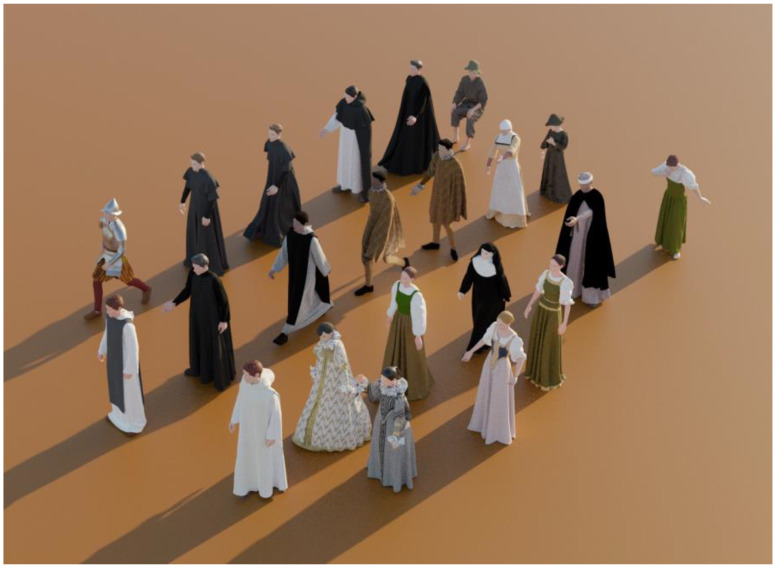
Characters used in the application.

The process of creating 3D character models involved the use of the program Marvelous Designer to design and create clothing. A custom avatar was first created with the MakeHuman program and then imported into Mixamo to add a human skeleton and movement. The clothing was designed by importing a pattern and redrawing it over the avatar, adjusting measurements and then virtually sewing the pieces together. To optimize the models for visualization, plane colors were used in simple clothing designs and complex designs were simplified by eliminating details and using image textures. Several characters were created, including knights, monks, nobles, and people from different social strata in the city, as seen in Figure 8 [59,65].

### 3.3. Tracking

To develop the tracking system, it is important to consider the selection of sensors that are suitable for the type of tracking required. In our case, as we need the location and orientation of the mobile phone, the selected sensors were the GNSS and IMU. It is also important to process tracking data from those sensors efficiently to obtain a real-time response. 

#### 3.3.1. Determination of Location

The GNSS positioning of the user is one of the features that will most affect the use of the application, so it is necessary to have a design that offers a fast and accurate interaction. To locate a point within the earth’s surface, it is necessary for it to have unique coordinates based on a reference system. The output of the GNSS positioning system is a set of geographical coordinates given in latitude and longitude values, along with an altitude measurement. To incorporate GNSS tracking in Unity, we employed an Input class that provides access to the location property that holds the device’s GNSS data.

To convert geographical coordinates (e.g., latitude and longitude) to the Unity coordinate system, we used the Universal Transverse Mercator (UTM) coordinate system, which uses meters as its unit of measurement and is commonly used for mapping and navigation. Therefore, it is necessary to transform geographic coordinates to the UTM, for which the .NET library CoordinateSharp [66] was employed. This library provides geospatial calculations and coordinate conversion capabilities for applications developed using the .NET framework and supports a wide range of coordinate systems, including UTM, MGRS or WGS84. However, CoordinateSharp function calls are costly for application performance. To solve this issue, these calls were only performed when the geographic coordinates of the current frame were different from those of the previous frame. 

Additionally, to work with more manageable coordinate values, the UTM coordinates of the center of the city model E: 371197.41 N: 3151901.76 were translated to the origin of Unity coordinate system.

**Figure 9:**
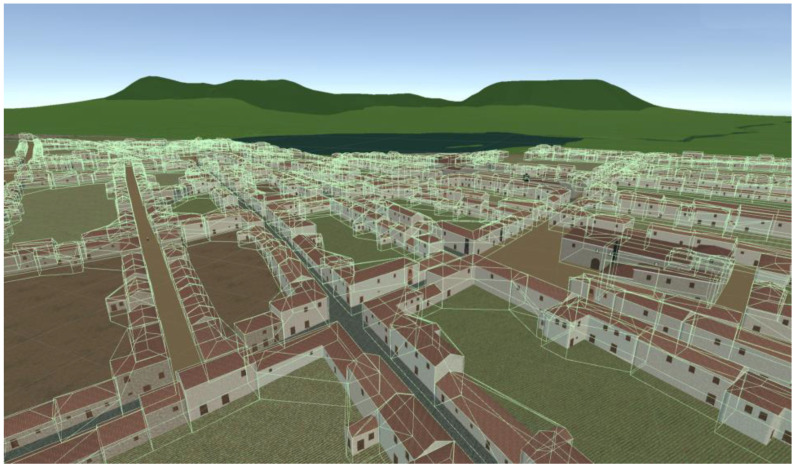
Convex mesh colliders (lines in green) adapted to the buildings in the city.

**Figure 10:**
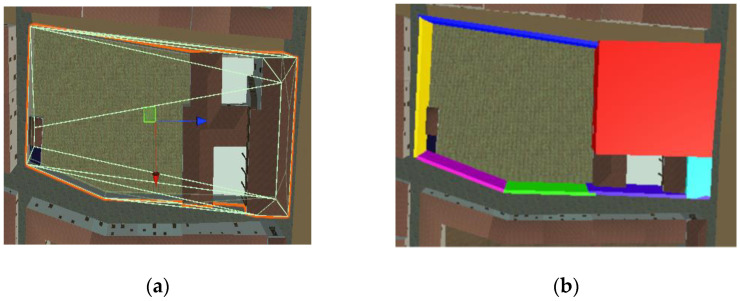
(**a**) Casa del Adelantado singular building viewed from above. In orange: outline of the building. In green: its convex mesh collider. Note the difference between them on the lower street. (**b**) Composition of Casa del Adelantado using boxes and box colliders.

One of the main problems with GNSS positioning is that it is not entirely accurate. By conducting some tests with mobile phones, it has been observed that the general average error of the horizontal and vertical coordinates in open field is around 5 m [67] and increases empirically in urban areas with the number of building floors: nfloors as 5+ nfloors meters. Although the number of floors in the historical center of the city is low, this is an important issue to solve. In case of an error in the GNSS coordinates, the user could be positioned inside a virtual 3D building. Several applications use other techniques in conjunction with GNSS to obtain more accurate data. For example, Google’s location services use nearby Wi-Fi networks, mobile networks, and other sensors on the same device [68]. Using those kinds of techniques would be complex and time-consuming, so a different approach was used to overcome this problem. To avoid the user being placed inside a model building, the proposed solution was based on establishing a collision system. In Unity, a collider is a component that is used to define the shape and size of an object’s physical boundary, which is used for collision detection with other objects in the scene. In this project, we used two types of colliders for almost all constructions: box colliders with the shape of a rectangular prism, and convex mesh colliders that adapt to the shape of the object. Using a convex mesh collider improves performance because it simplifies the shape of the collider. However, it can also lead to inaccuracies in collision detection because the collider may not match the shape of the mesh exactly. Convex mesh colliders adapt to most of the city constructions correctly, since they mostly have quite simple shapes (see Figure 9). However, a few singular religious and civil buildings have more complex shapes that cannot be fitted using only a convex mesh collider. In this case, the object was covered by box objects, each one with a box collider, that are fitted to the building (see Figure 10). 

After all these preparations, it is now possible to avoid transporting the user inside a building by first sending an object to the new coordinates that checks for a collision. If it has occurred, it can be confirmed that a building exists in that position, and the user will not be moved to the proposed coordinates.

Another problem with GNSS positioning is related to its vertical component. The location coordinates are typically 5 times more accurate horizontally than vertically [69]; therefore, a mean vertical error of 25 m is expected. To provide an accurate approximation of the altitude, we used a very common element in game development called a raycast.

**Figure 11:**
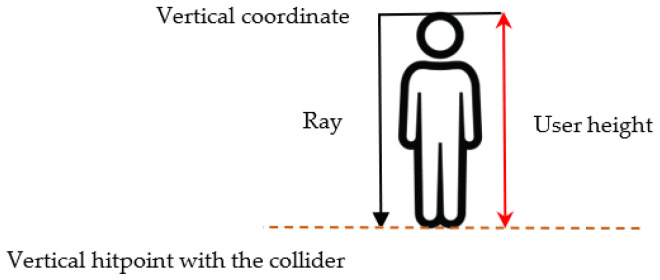
Determination of the vertical location of the user.

Raycasting refers to techniques that involve the launching of “rays” capable of colliding with surfaces and returning the set of objects with which it intersects along its trajectory. In Unity, these rays only collide with objects that have a collider component. Based on this collision, an action could be established through checks with the name of the object, its label, or other elements. To use this technique, a label called “Ground” was set for all the 3D objects the user can stand on, and a mesh collider was set for all of them. Then, when the user moves, a ray is launched pointing downwards and from the object that represents the user, obtaining the collision point with the collider of the “Ground” objects, as shown in Figure 11. 

This collision point is then used as the new vertical coordinate calculation using the following formula:

Vertical coordinate = Vertical hitpoint + User height(1)

The height of the user is a parameter that can be adjusted in the application. In this way, the height is changed progressively, considering the elevation of the terrain using the “Ground” label.

Finally, another problem to be solved is the GNNS sampling rate. Most modern smartphones and tablets have a default GNNS sampling rate of 1 Hz, which means that the device records the user’s location once per second. However, the effective sampling rate also depends on the computing power of the device and the processing speed of Unity. Since the updating of coordinates only occurs every certain time, the user may change their position to their new coordinates on the ground very abruptly. This has a negative impact on the user experience, as it is possible to become disoriented and confused. To improve this aspect, the moveTowards function in Unity was used. This function allows one to move an object smoothly from its current position to a new desired position with a predefined speed, and was used to model the movement of the user.

#### 3.3.2. Determination of Orientation

To enable users to explore and view their surroundings after locating themselves on the ground, the camera rotation in the application must match that of the user’s phone. To achieve this, the gyroscope of the mobile phone is utilized to obtain the current rotation data of the device. The Unity Input class provides access to the gyro property, which stores the device’s orientation data as a quaternion variable named attitude. In Unity, rotations are represented using quaternions, which are a mathematical method of representing rotations in a three-dimensional space [70].

**Figure 12:**
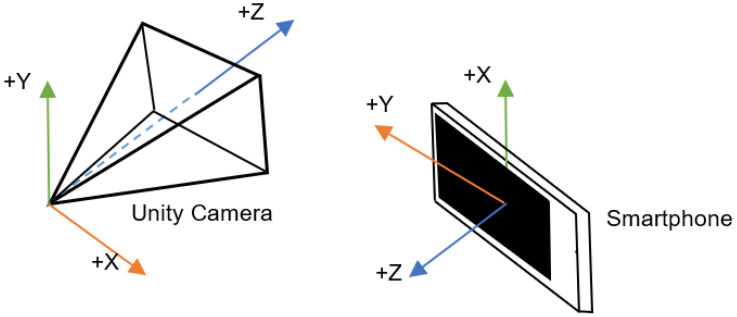
Difference between the coordinate systems for the smartphone’s gyroscope and Unity.

When reading gyroscope data from a smartphone in Unity, it is important to consider the difference in coordinate systems between the smartphone and Unity. The gyroscope in the smartphone is typically a right-handed coordinate system, while Unity uses a left-handed coordinate system (see Figure 12). Therefore, to properly use the gyroscope data in Unity, it is necessary to convert the attitude from the smartphone axis to the Unity axis [71].

### 3.4. Visualization

**Figure 13:**
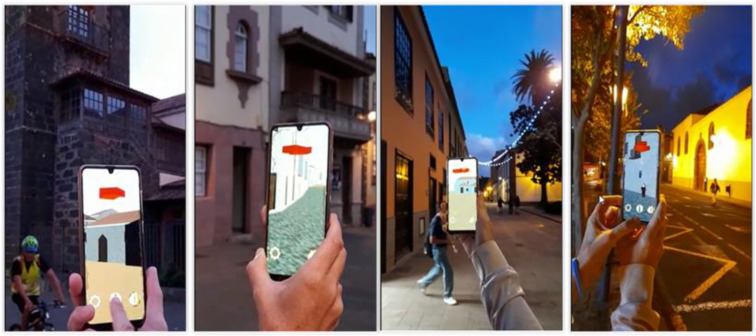
Several views of the application execution in different city points.

LagunAR was designed to visualize the 3D model of the city of San Cristóbal de La Laguna on a smartphone screen. At any given moment, the displayed model on the screen corresponds to the user’s real-time location, which is transported five centuries back in time (Figure 13). The transitions between views should be smooth as the user walks with the device, ensuring a satisfactory user experience. However, due to the limitations of the chosen platform for LagunAR, which include screen dimensions, computational capabilities of mid-range or high-end mobile phones, and the level of detail in the model, additional optimizations beyond tracking are necessary for the displayed views. The goal is to guarantee a frame rate of 60 FPS, as a higher frame rate results in smoother animation of the model and a more comfortable experience for the user. Performance requirements are critical, so it is necessary to minimize the resources used by the visualization. In the following subsections, we explain the optimization techniques that were implemented during development: occlusion culling, texture reduction, and the optimal selection of renderer distance from the observer.

The Unity 3D game engine renders each triangle in the scene within the view volume, discarding any polygons outside of it. However, regardless of the position of the camera or observer, all triangles inside the view volume or frustum are rendered, even if there are objects standing between the camera and potentially hiding them. In terms of computation, all objects within the view volume are visible to the observer. Occlusion culling is a technique that enables rendering to ignore objects that are not in the player’s view, saving valuable computational resources and reducing the workload on the CPU and GPU.

During the occlusion process, Unity divides the space into cells and generates a binary tree that contains information about the geometry within each cell and the visibility between adjacent cells. This tree structure is created during the baking process and is loaded into memory to efficiently determine which cells are visible from the camera in each frame during runtime. The two essential options for configuring occlusion culling are

- Smallest Occluder: this parameter represents the minimum size an object must have to block others, meaning that the object prevents the camera from seeing objects behind it.
- Smallest Hole: this parameter defines the minimum size of holes through which the camera can see.

**Figure 14:**
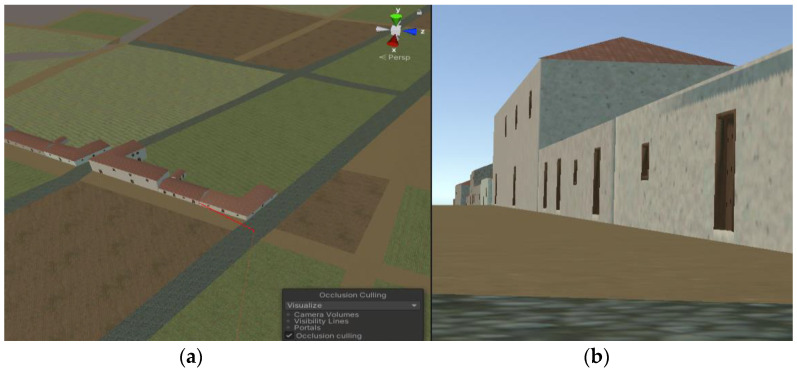
Example of occlusion culling: when looking at the right side of a street (as seen with the red ray in (**a**)) with buildings on both sides, only the buildings on the side you are looking at are rendered (**a**). Rendered houses are shown in (**b**).

It is important to strike a balance between these two parameters, since both options impact the quality of the result, the time required for computation, and the memory consumption. If the minimum sizes are smaller, more objects need to be considered, depending on whether the model contains numerous small holes or small objects. In the case of the San Cristóbal de La Laguna, there are not many small objects or holes, so the impact was not significant. Figure 14 illustrates the technique.

**Figure 15:**
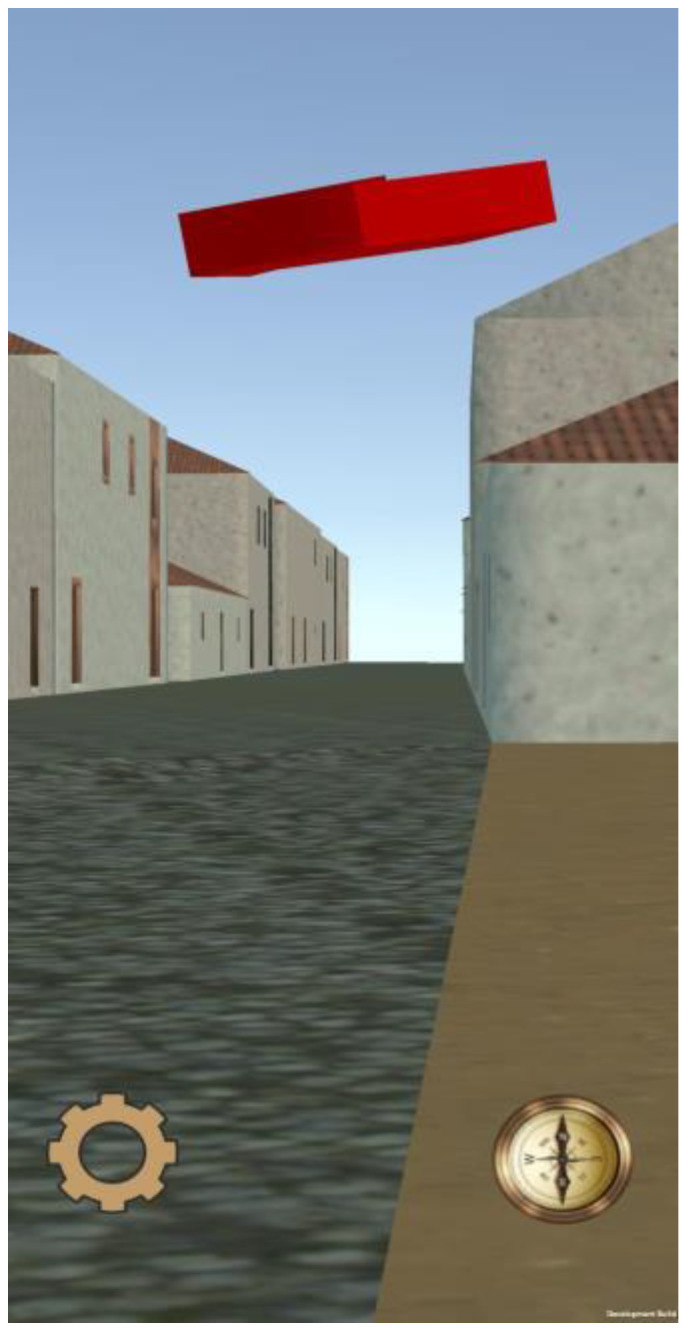
Result of reducing the render distance.

On the other hand, the render distance determines whether an object will be drawn based on its distance from the camera. Since smartphones, on which the application will run, have smaller screens compared to typical monitors, it is possible to reduce the rendering distance to some extent without significantly affecting the graphic quality (see Figure 15).

The 3D model of the city was procedurally generated with 3D CityEngine and later it was exported to Blender. The result of this process is a model made up of a single object that reproduces the historical center of the city of San Cristóbal de La Laguna. This version of the 3D city is not suitable for applying the occlusion culling technique, since with a single occlusion object, it is impossible to establish which buildings hide another in each camera view. This problem was solved by subdividing this object. Two options were tested: subdivision by square tiles and subdivision by individual building. This last option achieved an adequate frame rate results on the mobile and it was obtained by using a script that was run over the model in Blender. 

### 3.5. Interaction

To ensure that all the developed elements can be easily and effectively used, it is necessary to create a Graphical User Interface (GUI) that is intuitive for the interaction of the users with the application.

#### 3.5.1. Graphical User Interface

The application interface provides guidance to users as they explore the city, displaying the model of the ancient city on the screen and directing them through an arrow that indicates the direction of the next point of interest (POI) they should proceed to. Upon reaching the designated objective, relevant information about the building or character is automatically displayed. 

**Figure 16:**
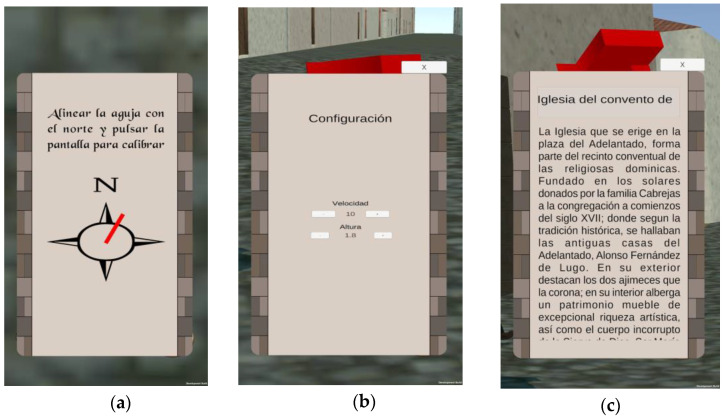
Smartphone screens: (**a**) manual calibration, (**b**) parameter configuration, and (**c**) POI information.

As explained in Section 3.2, the guidance phase relies on GNNS and IMU positioning calculations. To ensure that the IMU works for all kinds of smartphones, a manual calibration process was also provided. This process aligns the mobile device’s gyroscope with the magnetic north. During this phase, the user is prompted to align a needle with the magnetic north using a specially designed icon that consists of a compass rose, and a red needle representing the angle between the direction in which the top of the mobile device is pointing and the magnetic north (Figure 16a). 

The speed of the movement and the height of the user is also configurable. A menu was designed to customize these parameters based on user preferences. The data can be entered manually, but also increased or decreased with the buttons provided for it, as shown in Figure 16b. 

The application interface uses a single panel in which the texts of the POIs are dynamically loaded; the scrolling of the text will not automatically restart when the next one is loaded, since new text objects are not being created, but the object is modified by applying a new position for every scroll event. To work around this issue, the button that closes the panel also changes the offset value; therefore, the next text to load is at the initial position. This technique is also used for visualizing the name of the POIs, but adapting a horizontal scroll instead of a vertical one (Figure 16c).

**Figure 17:**
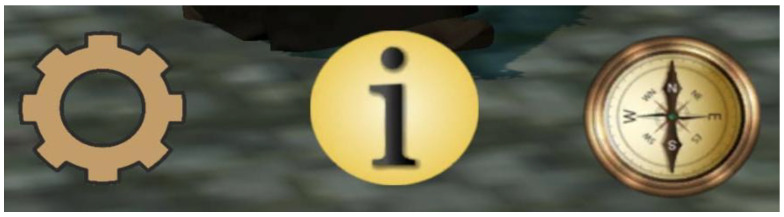
Graphic buttons for configuration, information and manual calibration.

Finally, it should be noted that in AR tracking mode, the user has access to each menu previously mentioned through graphic buttons, represented by self-explanatory icons regarding their purpose: configuration, information and calibration (Figure 17).

#### 3.5.2. Guidance System

The guidance system relies on a visual icon that is consistently overlaid on the scene view, indicating the direction the user should move towards to reach the nearest POI. Additionally, this system allows players to obtain information about any POI by simply pointing the smartphone at it.

A visual guidance system was designed, utilizing a 3D arrow as the chosen icon. The ArrowGuide class is responsible for updating the arrow’s rotation so that it points to the nearest unvisited POI, ensuring that the user is directed towards new locations and not the ones they have already explored. Upon initialization, the ArrowGuide class searches for and stores all the objects in the scene marked as points of interest in a list. Each point of interest in the list has a reference to a Boolean variable that tracks whether the player has read its associated information. The class also includes a method that calculates the closest unvisited point of interest.

**Figure 18:**
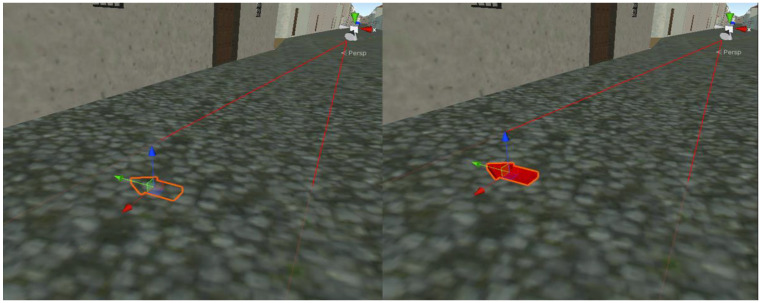
Model of guidance arrow before and after using the shader.

**Figure 19:**
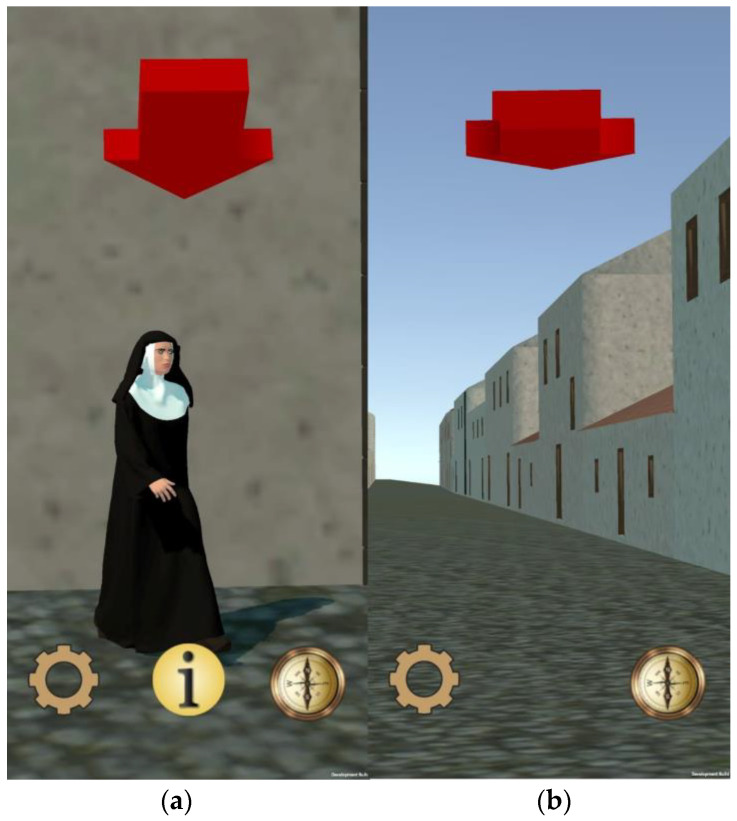
Screen capture where the arrow points to a POI (**a**) and directs towards a new POI (**b**).

To ensure the arrow is always visible in the camera view, a shader was employed to modify the color, light, and darkness levels of the city model. Specifically, the shader paints the arrow symbol in red, providing sufficient contrast against the predominant colors of the city model (Figure 18). The final result for the visual guidance system is shown in Figure 19.

### 3.6. AR System

A functional prototype of a mobile application was developed to guide visitors through the streets of San Cristóbal de La Laguna, showcasing the city as it appeared in the 16th century. Throughout the user’s experience, they walk through the present-day city while viewing its historical counterpart. The application aims to enhance the visitor’s understanding of the city’s rich heritage, emphasizing its key value of preserving the urban layout and its status as a Renaissance city. Simultaneously, the application presents the transformation of its buildings and provides information about the most significant structures. The key features and requirements of the application are described below.

#### 3.6.1. Class Structure and Relationships

**Figure 20:**
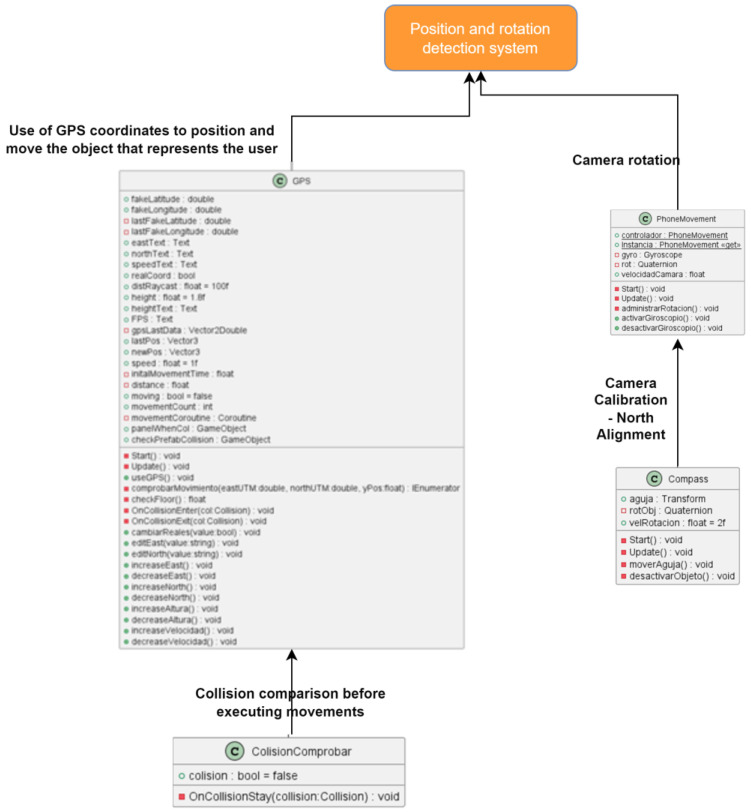
Most important classes in the user positioning and rotation system. (Source: generated by authors).

**Figure 21:**
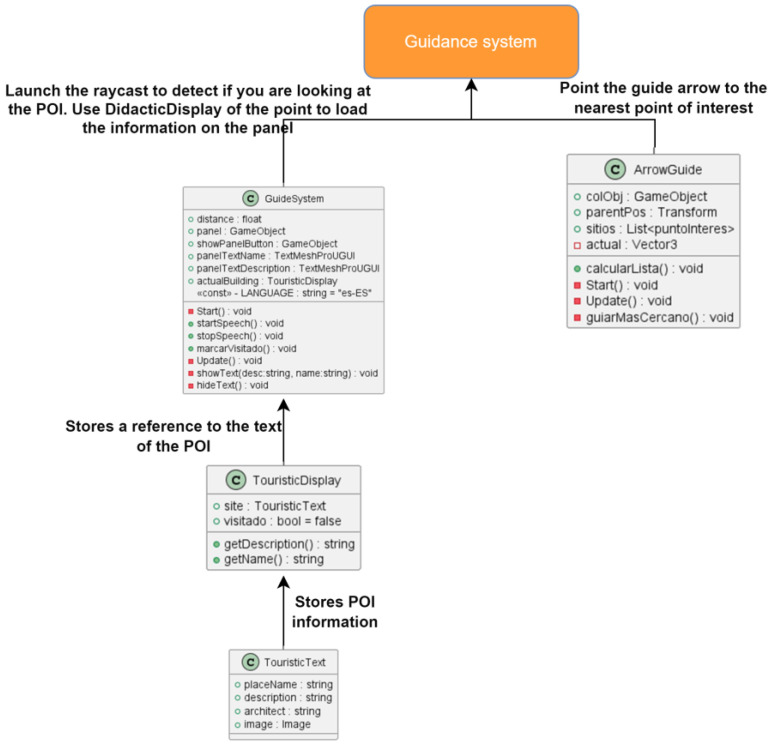
Most important classes in the user positioning and rotation system. (Source: generated by authors).

It is worth mentioning that the development has solved two key problems that we highlight in this section. On the one hand, we have the user’s positioning and orientation system in the georeferenced model. In Figure 20, we can see the main developed classes and their dependencies. This subsystem, in addition to managing GPS positioning, also implements functions related to device calibration. On the other hand, the guide system and information management of the POIs of the route stand out, which implement the main functionality of the route that the user follows. In this subsystem, the classes that manage the POIs, their information and key aspects in the visual guide, such as the redirection of the arrow, are implemented (Figure 21).

#### 3.6.2. System Architecture

The system’s development involved defining a set of requirements for the application, which are detailed as follows:

- The application has to display a georeferenced 3D model of the city as it existed in the 16th century, enabling users to explore it while moving through the real city;
- The application will seamlessly load the current visible landscape for the user as they navigate the city;
- The application will accurately convert GNNS coordinates into world coordinates within the scene to position the user correctly within the model;
- The application has to incorporate a system to represent buildings that no longer exist in the present-day city;
- The application will include a set of objectives to guide users to the next POI;
- Characters representing various social classes and professions from 16th-century Spain have to be included in the application;
- The application will provide a local information system, allowing users to access historical information about the specific POI they are focusing on;
- The application will utilize the mobile device’s sensors and handle data persistence on the device itself.

**Figure 22:**
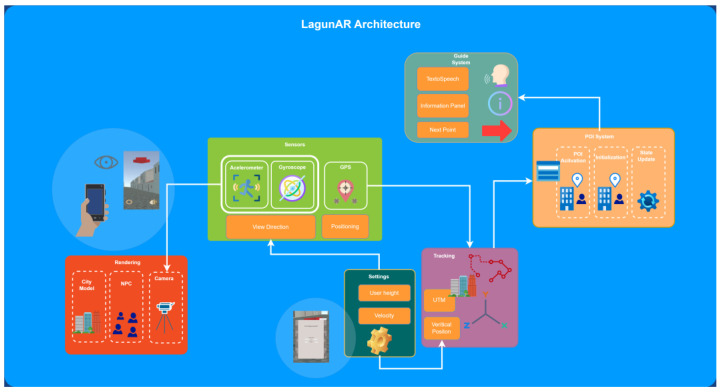
System architecture.

Given the previous requirements, the architecture of the application is shown in Figure 22. The application includes the guidance system, responsible for communicating the POIs to the user along the route. This communicates with the POI system, which initializes, activates and updates them. The computations that track the user’s journey at all times require the sensors and tracking subsystems, which obtain the parameters for their execution from the initialization subsystem. The POIs system, in turn, needs the information generated at every moment by the tracking subsystem. Finally, the rendering subsystem generates the views of the 3D model by orienting the virtual camera according to the view that the mobile camera would provide, obtaining the data from the sensor subsystem.

#### 3.6.3. Information System 

As explained in the previous section, the application has to provide an information system allowing users to access historical information about the city POIs. This information was obtained from the catalog of Cultural Assets provided by the Cultural Manager of San Cristóbal de La Laguna, developed by the International Center for Heritage Conservation Foundation (CICOP). The Cultural Manager is a web application that contains extensive information such as historical background, architectural description, location, and images related to the POIs. The information associated with each point of interest is stored using Unity Scriptable Objects to ensure efficient and fast access when the user activates it in the view. The TouristicDisplay class handles the persistence and stores references to the Scriptable Objects for each POI. To detect interaction with a visible point, a raycast is used in the view direction. It checks if the ray intersects with a building using its label, which also has an associated TouristicDisplay component. If both conditions are met, the reference to the associated ScriptableObject is retrieved, and the information is displayed in the text panel when the player presses a button on the screen. It is important to note that new information is only loaded if the previous information panel is not visible, preventing multiple panels from overlapping.

The information loading process was semi-automated using a tool designed for the Unity Scene Editor (Figure 20). This tool allows the location of buildings on the game map using UTM coordinates, which correspond to the geographic coordinates provided by the CICOP. It is worth noting that some buildings in the catalog may not exist or have undergone transformations compared to the map. In such cases, an object is assigned, including an icon representing the site, a collider, a label, and the corresponding script, allowing the designed interaction system to be applied similarly to other points of interest.

## 4. Results

In this section, we present some results for the AR application in 3D modeling, tracking, visualization and interaction.

### 4.1. 3D Modeling

**Figure 23:**
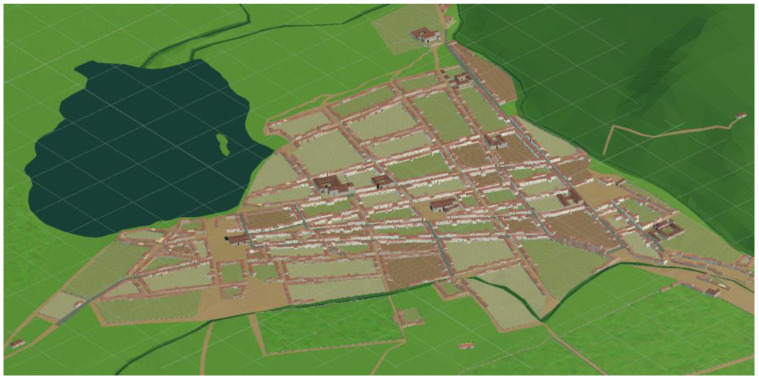
A 3D model of the city.

The techniques presented in Section 3.1 were combined to generate the complete 3D model of a city. As shown in Figure 23, the model spans approximately 15 km^2^ and contains around 1300 houses and 16 singular buildings. The 3D reconstruction demonstrates the successful generation and optimization of visually appealing and performance-efficient 3D models suitable for mobile applications.

### 4.2. Tracking

**Figure 24:**
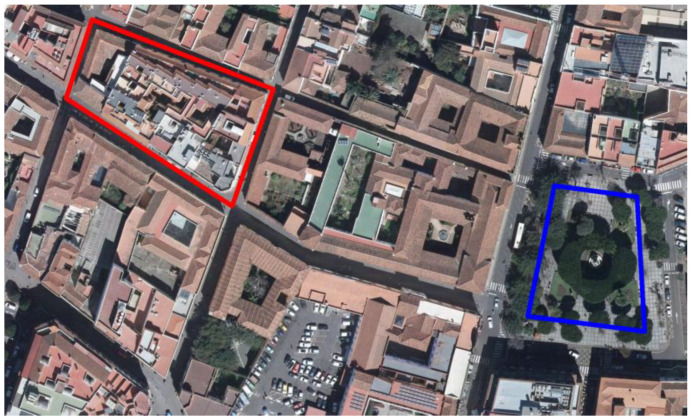
Test circuits for tracking precision. Square circuit shown in blue and block circuit shown in red.

To evaluate the location and angle precision of the mobile AR application, a set of experiments were conducted. We selected two circuits in the city, one in an open square show in blue in Figure 24 and the other in red surrounded a block with buildings in the historical center as shown in red Figure 24. The mobile phone used in the experiments was the mid-range smartphone Xiaomi Redmi Note 11S.

**Figure 25:**
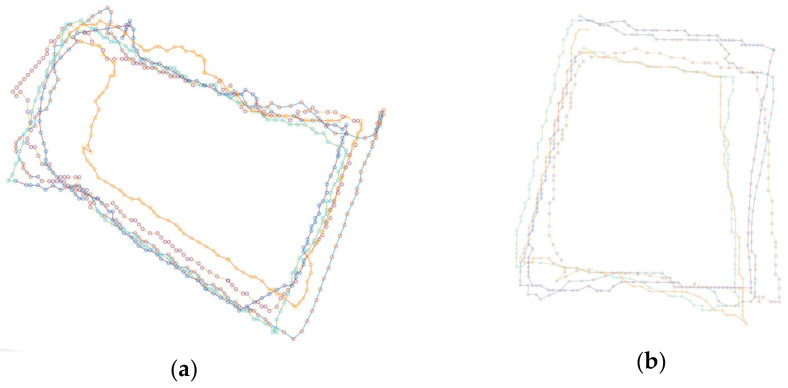
GPS point location over the block (**a**) and square (**b**) circuit. Each loop is shown in a different color.

The location precision of the mobile AR application was evaluated by following a closed path in each circuit. Several samples were taken in several loops in the circuit, recording the location positions through the circuit. To estimate the errors, we took a cross-validation approach. For each loop, its positional measurements were used as the training set (and therefore used as the estimation of the true location on the loop) and compared with the rest of the loops (used then as a test set). The locations for both experiments are shown in Figure 25.

The average distance error and standard deviation, percentiles of 25%, 50% and 75% of the error (Q_1,_ Q_2,_ and Q_3_, respectively) and maximum distance observed in the experiments are shown in Table 1. Distance errors did not consider the height error.

**Figure 26:**
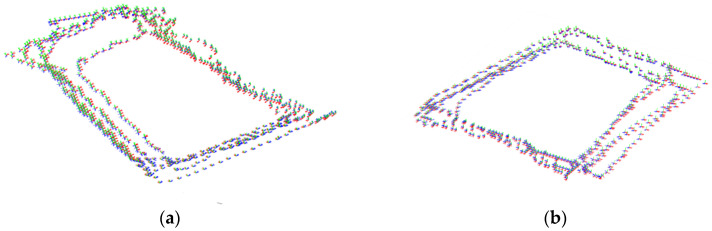
Angular variation over the square (**a**) and block (**b**) circuit. The Z axis of the smartphone is shown in blue while the X and Y axes are show in red and green, respectively.

The angle precision of the mobile AR application was evaluated by measuring the deviation of the Z axis of the smartphone (see Figure 26) between different loops around the circuits. Note that the measured angular deviation is not the intrinsic angular error of the smartphone. Instead, it measures the angular deviation due to the movement of the user. The angle changes observed in the experiments are shown in Table 2

Qualitative results are shown in Figure 26 for the block and square circuits. The Z axis of the smartphone is shown in blue while the X and Y axes are shown in red and green, respectively.

### 4.3. Visualization

**Figure 27:**
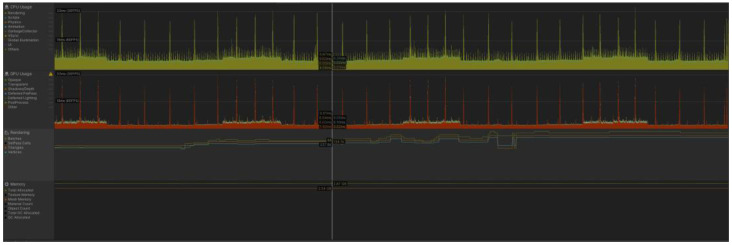
Unity profiling of the application.

We evaluated the performance of a mobile AR application developed with Unity on a mid-range Xiaomi Redmi 11 smartphone. It is equipped with a Mali-G52 MC2 GPU developed by ARM, which is commonly used in smartphones and other mobile devices. Specifically, we measured the frame rate, CPU usage, GPU usage, triangle count, and memory usage of the application using the Unity Profiler tool (see Figure 27).

Frame Rate:

The frame rate of the mobile AR application was found to be higher than 60 frames per second (FPS) throughout almost all of the testing period.

- CPU and GPU Usage:

The average CPU usage of the application was measured to be around 9 ms. per frame, while the GPU usage was measured around 5 ms. per frame.

- Triangle Count:

The triangle count of the application depends on the number of characters being displayed and was measured to be around 130,000 for a scene without characters to 750,000 when they are displayed. The typical number of rendering batches was measured around 75.

- Memory Usage:

The memory usage of the application was measured to be around 3 Gb and is mainly dedicated to store the mesh. 

### 4.4. Usability

The analysis of the results obtained cannot ignore the evaluation of the usability of the application. In our case, testing was used to detect design problems, so we have chosen a heuristic evaluation and also a qualitative evaluation based on tasks with which the users probe the application. 

#### 4.4.1. Heuristic Usability Tests

A valid alternative to the tests with users is heuristic tests carried out by experts to anticipate usability problems [19]. In line with this, Nielsen’s 10 Golden Rules or Shneiderman’s 8 Golden Rules are widely accepted as standards to comply with by web and mobile applications. However, when it deals with AR applications, there are aspects of this type of applications related to interaction that we must check. Specific proposals for testing usability in AR applications can be found in [72,73]. These two papers extend the 10 Nielsen principles; this is the reason we evaluated both of them, as shown in Table 3 and Table 4. They show each characteristic to be analyzed and the observation made by the expert.

#### 4.4.2. Qualitative Testing with Users 

This test was designed to detect usability issues before involving development on a larger scale. User evaluations are known to reveal usability problems in applications, but also it has been empirically established that five users detect approximately the 80% of problems, and eight users detect 90% [74]. Following the Nielsen recommendations, the use of the application was tested by five users with no prior knowledge about it. A pre-test was designed, with questions about the characteristics of the user focused on their knowledge of the technology. The selected users have little or no knowledge about Augmented Reality but normally use their mobile phone. They are travel-loving users who are interested in the historical and cultural heritage of the places they visit, aged between 17 and 47. The selection criteria for users was focused on potential users, corresponding to people with an interest in history and cultural heritage. The group of users have the profile Archeologist in ACUX [57]. With the objective of detecting design mistakes, it also was important to have people who knew the city, in order to carry out the tour more easily, so they were able to give their attention to the application more than to the POIs. Likewise, a post-test was included that has the questions on the SUS Usability scale [75] as well as an open question to indicate any appreciation that seems relevant to improve the design of the application. Although the results of the SUS test cannot be considered significant, they have been included as a guide. Due to the interaction in the application is based essentially on the user’s geo-position, there is an extra difficulty for testing: they cannot carry it out in a usability laboratory, but they must do it in situ. 

**Figure 28:**
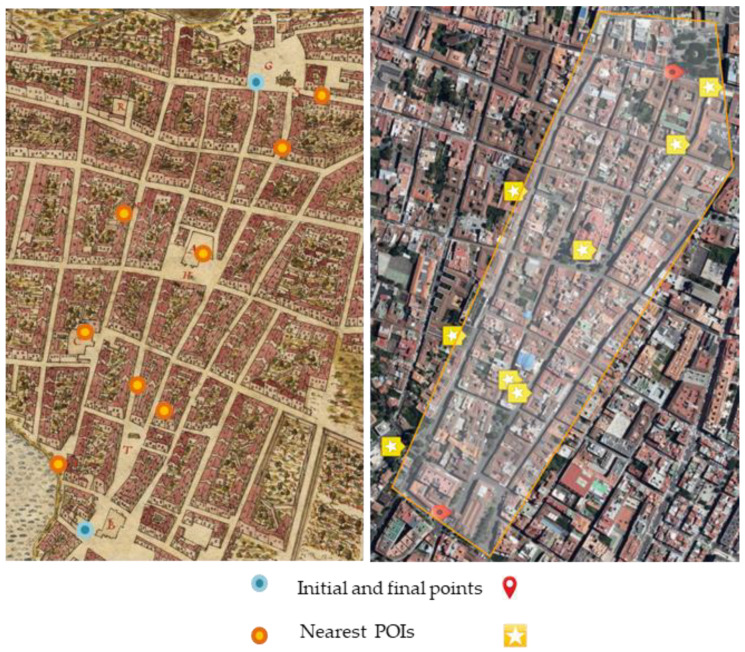
User’s test: initial, final and nearest POIs in the ancient and current map.

The test consists of a task based on a scenario where the facilitator accompanies the user, recording their reactions and interactions on the screen, as well as both pre-test and post-test questionnaires. The task indicates a starting point and another ending point in the historic center of San Cristóbal de La Laguna, along which it will find points of interest, so the UI will activate the elements designed for interaction. The recommendations of Nielsen [72] for the elaboration of usability tests in Augmented Reality applications have been taken into account: it should be considered that the participants may need some guidance in the initial steps of the application, the download times of the application may be longer than usual and the test may require more time than is normally used in conventional applications. The user’s experience was unrestricted, and they were making decisions about their destination each time. Figure 28 displays both historical and contemporary urban areas. Over the two maps have been marked the initial and final points and also the POIs, probably where the users would be near. This area includes the three main streets in the historical center of San Cristóbal de La Laguna. In the current map, the most probable area for the user’s trip is shaded. This polygon has about 700 m of large from the initial point to the final point in the task, and approximately 250 m. Before the users started the route the facilitator explained in a short form the purpose of the application. Also, the facilitator asked the users to tell all their thoughts and every question or issue related to the application. This was remembered to the users several times along the tour. The task was developed in a pedestrian zone, so there were few dangerous situations. For more security, the facilitator notified them of the proximity to intersections, with some not pedestrian streets or some obstacles in the route.

The metrics used were time taken to perform the test, completeness of the test in terms of the number of reachable POIs visited, number of users that finished the test, number of times users had to press the back button on their mobile, number of interactions without effect (camera zoom, arrow tap, teleport), and number of questions carried out to progress on the task. Initially, users were asked to install the application, but some did not have mobiles with the necessary requirements to run it, so it was decided to always perform the test with the same mobile: a mid-range Samsung Galaxy A31 with Android version 12.

Overall, users find the app easy to use and suitable for both: the design of elements and layout. However, they feel frustrated when the compass does not work correctly. Over and over they select points on the screen to focus the view on, and they are frustrated when GPS precision guides them to places slightly off-set from buildings. To solve this problem, some users repeatedly try to zoom in and out with a gesture on the screen, and some say they would like to have a zoom function, especially in narrow streets. The arrow icon is recognized as a direction to go, but users associate it with an interactable object, clicking it over and over again, especially at the beginning of the test. Users identify that the information icon must be clicked, but sometimes it is instantly displayed as on and off without the user being able to click on it. Also, variability with GPS performance is detected depending on the climatology: the test developed on days with calima (sand and dust particles from the Sahara Desert transported by winds) stopped the GPS more frequently than in a normal day. A user proposes adding images of historical monuments on the screen, also an option without a route, independent of GPS. This is because the failures of the GPS make it necessary to pay more attention to the calibration of the device than to the experience of the journey. Text-to-speech needs improvement; for example, centuries in Roman numerals do not read correctly. If the POI name overflows the panel, it is hidden and some users ask how they can see it. In general, users feel comfortable and have no difficulties when GPS provides accurate information because the arrow is correctly oriented and the scene matches the environment. However, when the accuracy drops, the experience is not as satisfying.

**Figure 29:**
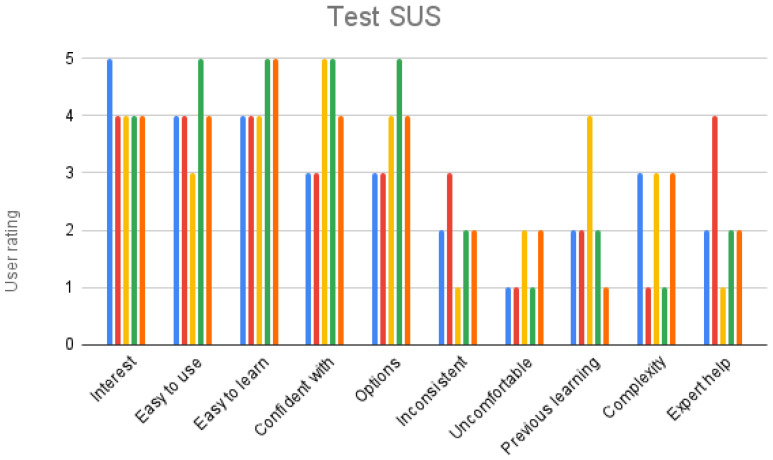
User responses to the SUS test (each color represents a user).

Regarding the responses to the SUS test, they ratify the visual observation, obtaining average or high scores in terms of the positive aspects and low-medium, observing the need to provide some way of prior learning or support for use (Figure 29).

Regarding the metrics, the interactions that show frustration stand out: number of times help is requested, and the number of interactions without effect in which an attempt was made to resolve situations perceived as problematic throughout the tour. Help was requested by some users on a maximum of three occasions during the journey, which on average was about 25 min long. The most recurring option among users when faced with the problems they encountered was the attempt to zoom in or out of the camera or click on some point on the screen, hoping to solve the problem in one way or another; on average this happened 2.5 times every 10 min. All users completed the task without pressing the back button; however, there was variability in the number of points visited.

In view of these results, the following modifications are proposed to obtain a better design. A brief descriptive explanation must be included in the initial screen of the application: it must include that the horizontal position is required to calibrate the compass and that the arrow does not allow interaction. For this, the intensity of the arrow material will be attenuated as a disable message. In this introduction, the screen would include the type of recommended device and an announcement of the loading status and loading time (26”) of the models. In relation to the route, a delay of milliseconds should be included in the display of the information icon, and a zoom-in–out event associated with some gesture on the screen should also be implemented. 

## 5. Discussion

The results in Section 2 and Section 3 show how the LagunAR application provides a reconstructed 3D model of the city in the 16th century that is shown on a mobile phone over the actual city using geolocation. The geolocated position is used also for providing information of several points of interest in the city and its inhabitants.

The evaluation of the location and angle precision of the mobile AR application was conducted through a set of experiments in two different circuits within the city. The circuits included an open square and a block surrounded by buildings in the historical center. The tracking precision of the mobile AR application was assessed by following a closed path in two city circuits and recording the positions at several points along them. The results indicate that the precision of the mobile AR application falls within acceptable limits for most use cases. The average distance error for the square circuit was found to be 3.63 m, with a standard deviation of 0.28 m. Similarly, the block circuit exhibited an average distance error of 3.88 m, with a standard deviation of 0.68 m.

The experiments suggest that the accuracy of the tracking system in the city is affected by various factors, such as the quality of the GNNS signal, the user’s location, the presence of obstacles like buildings and the meteorology. Despite these factors, the observed distance errors usually remain within acceptable ranges.

In addition to evaluating the location precision, the angle precision of the mobile AR application was assessed by measuring the deviation of the Z axis of the smartphone between different loops around the circuits. The measured angular deviation represents the user’s angular movement rather than the intrinsic angular error of the smartphone. The mean angular deviation for both the square and block circuits was around 32.5 degrees, with standard deviations of 5.91 degrees and 4.53 degrees, respectively. The results demonstrate a moderate angular variation across the samples and consistent estimation of the Z axis.

The evaluation of the location and angle precision in the mobile AR application contribute to the understanding of the application’s performance and provide valuable insights for further improvements in the tracking system.

The performance of a mobile AR application developed with Unity was evaluated for a mid-range smartphone. Various performance metrics were measured using the Unity Profiler tool, including the frame rate, CPU usage, GPU usage, triangle count, and memory usage.

The frame rate of the mobile AR application was consistently stable over 60 frames per second (FPS) throughout the testing period. This indicates that the application delivers a smooth user experience without significant lag, ensuring a visually pleasing and interactive AR environment. 

Regarding CPU and GPU usage, the average CPU usage per frame was measured to be around 9 ms, while the GPU usage was approximately 5 ms per frame. These measurements indicate that the application does not heavily burden the smartphone’s CPU, leaving sufficient processing power available for other tasks. The GPU usage is also relatively low, implying efficient utilization of the GPU resources. This combination ensures that the application runs smoothly and does not strain the device’s hardware capabilities.

The triangle count, which depends on the number of characters displayed in the application, was measured to range from approximately 130,000 for scenes without characters to 750,000 when characters are displayed. This suggests that the application utilizes a moderate number of triangles for its graphics, obtaining a good balance between visual quality and performance. Additionally, the typical number of rendering batches was measured to be around 75, indicating efficient rendering optimizations.

In terms of memory usage, the application was found to consume around 3 GB of memory, primarily dedicated to storing the mesh, indicating that the application utilizes a reasonable amount of memory and should not lead to memory constraints or performance slowdowns. 

These results suggest that the application can deliver a smooth and engaging user experience on a wide range of smartphones with similar or higher specifications.

Once the technical aspects of the application were discussed, we proceeded to detail the elements related to the usability of the application. As explained in Section 3, this can be approached using three main categories: user testing, qualitative tests, and heuristic expert’s evaluation. In our case, user testing and heuristic evaluation were chosen.

Regarding heuristic evaluation, the usability expert assessed the main elements of the AR application. There are several positive elements to emphasize. The design of the interface using icons that effectively communicate their function. The consideration of accessibility in the interface that enhances visibility and usability. A user-friendly physical interaction that allows an intuitive navigation and exploration of the virtual environment. Areas to improve include occasional misalignments between the physical and virtual worlds and a better consideration of the user’s physical and perceptual abilities. Functional aspects of the application were also evaluated and showed several positive aspects such as smooth execution, consistency in interface design, informative audio elements, and georeferenced historical representations. The expert suggested improving the clarity of the initial loading screen and addressing occasional tracking-related issues. 

Finally, user testing was conducted with five users who had no prior knowledge of AR technology. They were asked to complete a task while their interactions and feedback were recorded. Metrics used included completion time, completion rate, number of back button presses, ineffective interactions, and questions asked to the facilitator. Users found the application easy to use, but they encountered occasional problems when tracking accuracy was insufficient. They suggested improvements such as zoom functionality, clearer instructions, and improved text readability.

In conclusion, the evaluation results show a positive impression and highlights the areas for design improvements in the application. These include clarifying instructions, addressing tracking-related issues, improving text readability, and implementing zoom functionality.

Our approach presents several advantages compared to other AR applications:

1. City-Scale reconstruction: LagunAR offers a city-scale reconstruction of the historical center, setting it apart from existing AR heritage applications that focus on individual sites or specific historical elements. This comprehensive approach allows users to explore an entire historical city, providing a broader perspective on the heritage site.
2. Enhanced engagement: LagunAR’s city-scale reconstruction significantly enhances engagement compared to other AR heritage applications. By immersing users in a reconstructed 3D model of the entire historical city, it fosters a deeper connection with the historical context.
3. Technical performance: LagunAR’s equals the technical performance of many AR heritage applications, even at a city scale. It maintains a stable frame rate, ensuring a smooth and enjoyable user experience throughout the extensive historical city.

This approach has also to face several challenges:

1. Tracking accuracy challenges: While LagunAR offers a city-scale reconstruction, it still faces the common problem of tracking accuracy. Factors like the quality of GNSS signals and environmental obstacles can affect tracking accuracy. Addressing these challenges is a shared concern in the AR field, especially when working with extensive reconstructions.
2. Limitations in usability and user feedback: LagunAR’s usability assessment, while positive, still reveals some challenges related to tracking accuracy and the sheer complexity of a city-scale reconstruction. Improvements in areas like text readability and zoom functionality have to be addressed when dealing with extensive reconstructions.

## 6. Conclusions and Future Works

Our paper described in detail the development of an AR application to recreate a 3D model of the historical center of San Cristóbal de La Laguna, where the users transverse the area. Along the route, points of interest in the city are shown and the user can obtain in-formation about them. The different subsystems that were implemented are explained, including the tracking system, sensor management system, parameter configuration system, Points of Interest (POIs) system, and information persistence system. Additionally, we described the rendering system and the accompanying 3D model design. During the implementation process, we took into account various considerations to address challenges associated with visualizing a detailed 3D model in real-time and ensuring synchronization with the actual surroundings. To achieve a more realistic model of the city in 1588, which no longer exists, the 3D reconstruction includes characters, clothing, bridges, fountains, religious crosses, water conduction system and walls. Clothing, social characteristics, and urban elements are designed following historical studies with the professional advice of the historian team member as well as singular buildings and the morphology of city houses. All these elements enhance the 3D content, bringing it closer to a more credible experience for the user.

Notably, we encountered difficulties in implementing the tracking system, particularly in achieving precise synchronization with the user’s location determined through mobile sensors. We also highlight the importance of maintaining a seamless progressive view of the model while users navigate the city. To reach the performance that is expected in this kind of application, optimizations typical of 3D applications in real-time were applied, as well as strategies in the tracking algorithm, limiting the computations in the update at each moment of the tour. 

Furthermore, the design of the User Interface adheres to simplicity principles and established interaction standards for mobile applications, ensuring usability. The accomplishment of the objectives was contrasted with different performance tests, measured by the number of frames per second when the application is run on a mid-range mobile, as well as the precision concerning the 3D model that is rendered in the mobile phone. Finally, it exposed usability analysis of the application, not only by a heuristic evaluation but also with qualitative tests with users. It was proven that the application satisfies the main usability attributes, but some specific problems were detected regarding the feedback provided to the user in critical situations and the zoom functionality. The main source of frustration for users was synchronizing errors between the virtual and real view. To further enhance location accuracy in urban environments, future research will focus on implementing advanced map matching algorithms. These algorithms will leverage GNSS data and road network information to align GNNS trajectories with the most probable routes taken by users. By considering road geometry, connectivity, and spatial constraints, map matching may effectively correct GNNS errors and provide more precise location information. 

Both the solutions provided in the implementation and the conclusions obtained in the usability study can be a useful orientation for the development of AR outdoor applications with the rendering of georeferenced 3D models. Our research contributes to the broad understanding of implementing and testing AR applications to create outdoor experiences at the city level. This work describes a set of useful practices to make AR applications using heavy 3D models widely available thanks to implementing a tracking system based on mobile phone sensors and optimizations to obtain quality 3D-content playing on mid-range mobiles. This benefits not only heritage AR applications but also tourism, and outdoor games or similar, at the level of whole-city models.

## Figures and Tables

**Figure 3:**
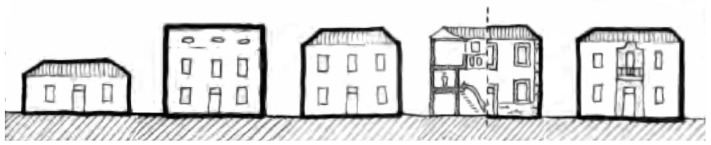
Typologies of houses in San Cristóbal de La Laguna (extracted from [59]). From left to right: terrera, granero, sobradada, comercial y armera.

**Table 1:** Location errors of the experiments.

Circuit	Mean Distance Error	Standard Deviation	Q_1_	Q_2_	Q_3_	Maximum Distance Error
Square	3.63	0.28	3.34	3.79	3.84	3.86
Block	3.88	0.68	3.37	3.61	4.31	4.97

**Table 2:** Angular change in the experiments.

Circuit	Mean Angular Change	Standard Deviation
Square	32.5 degrees	5.91 degrees
Block	32.4 degrees	4.53 degrees

**Table 3:** Expert assessment according to usability heuristics in [72] for Augmented Reality applications. On the left each of the characteristics to be analyzed in the application and on the right the observation by the expert is made.

Fit with user environment and task.	
Use visualizations and metaphors that have meaning within the physical and task environment in which they are presented.	The aesthetic of the User Interface (UI) objects is related to their functionality and it is inspired by the historical period which is represented: the wind rose, compass, arrow, …
Form communicates function.	
The form of a virtual element should rely on existing metaphors that the user will know in order to communicate affordances and capabilities.	Arrow guides to nearest POI, use of standard icons for setting and information.
Minimize distraction and overload	
Designs should work to minimize accidental distraction due to designs that areoverly cluttered, busy, and/or movement filled.	In addition to the view update with the user movement, the only element that changes is the visibility of the info icon and it does not do so in an intrusive way.
Adaptation to user position and motion.	
The system should adapt such that virtual elements are useful and usable from the variety of viewing angles, distances, and movements that will be taken by the user.	The view of the application is always adapted to where the user focuses, that is, where he directs his view.
Alignment of physical and virtual worlds.	
The placement of virtual elements should make sense in the physical environment. If virtual elements are aligned with physical objects, this alignment should be continuous over time and viewing perspectives.	The view of the application is always adapted to where the user focuses, that is, where he directs his view. Mismatches may occur depending on GPS accuracy.
Fit with the user’s physical abilities.	
Interaction with AR experiences should not require the user to perform actions that are physically challenging, dangerous, or that require excess amounts of coordination. All physical motion required should be easy.	The user will move in a pedestrian zone, he only walks and focuses the mobile with a gesture similar to taking a video. May be dangerous situations at junctions with traffic, but the application doesn’t show any warning.
Fit with user’s perceptual abilities.	
AR experiences should not present information in ways that fall outside of an intended user’s perceptual thresholds. Designers should consider size, color, motion, distance, and resolution whendesigning for AR.	The application has large icon sizes, the contrast is enough, however, the text in the information panels could present difficulties due to the font size. The view of the application is always adapted to where the user focuses, that is, where he directs his view.
Accessibility of off-screen objects.	
Interfaces that require direct manipulation (for example, AR and touch screens) should make it easy for users to find or recall the items they	Interface elements that the user interacts with, as well as interface elements that are informational, are superimposed on the view at all times.
Accounting for hardware capabilities.	
AR experiences should be designed to accommodate the capabilities and limitations of the hardware platform.	The application has been developed optimizing the models and checks at all times that the number of frames per second does not drop, the fluidity is checked. However, the initial load requires a wait time.

**Table 4:** Expert assessment according to usability heuristics in [73] for Augmented Reality applications. On the left, each of the characteristics to be analyzed in the application, and on the right the observation is made.

Functionality	
Application accommodates with the surroundings	The app uses GPS for updating the view of the scene to the actual environment the user is in, sometimes mismatches may occur depending on GPS accuracy.
Users are rewarded and rewards are meaningful	Does not apply
Application features do not stagnate	The app runs smoothly, fluently. The initial loading screen needs an element that informs the user about the status because it is not possible to distinguish if it is in process or stopped.
Application features are consistent	The same type of information panels are used throughout the application, the same type of feedback is applied and the same interface icons are used.
Audio-visual representation supported	The application has informative audios.
Accuracy of application features	The virtual world is georeferenced, it only depends on the GPS errors and precision. The historical models are a representation of the City in the 16th century made with the advice of an expert historian.
Effectiveness to achieve outcome	The application is reliable in the route and in the information, but sometimes the GPS has problems. It also has hardware dependency, the device must meet minimum requirements.
Application features are efficient to learn	The app has a simple interface with clear icons indicating the purpose of the interaction. The adjustment panel is simple, but the effect of the parameters must be explained.
User Friendliness	
User understands the functionalities	The interface elements are simple, just tap the information icon and follow the arrow. Configuration tasks are also required to calibrate the compass. All actions are self-explanatory because standard icons are used.
User does not have to memorize things unnecessarily	The application does not require memorizing the steps for any of the possible interactions.
User is in control	The user chooses at all times the actions to be performed except on the loading screen.
First-time experience is encouraging	The attractiveness of the virtual world generates interest in users, the first interaction may require feedback to improve the user experience.
No boring or repetitive tasks	The application objectives are achieved in at most two easy steps.
Application prioritizes user-satisfaction	The application has been designed to guide the user on a historical virtual route through the city in a pleasant way.
Application maintains relationship between real world and virtual objects	The application updates the virtual world at all times to adapt it to the real world in which the user is, mismatches may occur due to GPS inaccuracies.
Multiple operations not needed for performing simple task	The scene changes continuously according to the user’s position and updates the interface giving interaction options depending on whether it is in configuration, calibration, or guide mode.
Feature’s output does not match user expectation	In addition to the position change, the tasks to perform are very simple, all of them need at most two sequences of actions.
Design	
Application layout is efficient, simple and visually pleasing	As long as it is used with a mid-range or higher mobile device
Navigation is consistent, logical and minimalist	Yes
Icons are consistent and follow standard conventions	Yes
Application contains skippable non-playable content	Does not apply
Support	
Application gives feedback on the user’s actions	No
Application contains help and documentation	Application is self-explicative, it hasn’t any help or documentation.
Application handles errors	The application allows the calibration of the compass at all times and warns of failures that occur in the GPS, returning to a functional state when it is recalibrated.

## Data Availability

The test participants agreed with the use of the anonymously collected data for the users test. Data will be made available upon reasonable request to the corresponding author. The data are not publicly available due to privacy issues.
